# MiRKAT-S: a community-level test of association between the microbiota and survival times

**DOI:** 10.1186/s40168-017-0239-9

**Published:** 2017-02-08

**Authors:** Anna Plantinga, Xiang Zhan, Ni Zhao, Jun Chen, Robert R. Jenq, Michael C. Wu

**Affiliations:** 1 Department of Biostatistics, University of Washington, 1705 NE Pacific Street, Seattle, Washington USA; 2 Public Health Sciences Division, Fred Hutchinson Cancer Research Center, 1100 Fairview Ave N, Seattle, Washington USA; 3 Department of Biostatistics, Bloomberg School of Public Health, Johns Hopkins University, 615 N Wolfe St, Baltimore, Maryland USA; 4 Division of Biomedical Statistics and Informatics, Department of Health Sciences Research, Mayo Clinic, 200 First Street SW, Rochester, Minnesota USA; 5 Departments of Genomic Medicine and Stem Cell Transplantation, Division of Cancer Medicine, The University of Texas MD Anderson Cancer Center, 1515 Holcombe Blvd, Houston, Unit 1954 TX USA

**Keywords:** Survival data, Kernel machine regression, Distance-based analysis, Community-level analysis

## Abstract

**Background:**

Community-level analysis of the human microbiota has culminated in the discovery of relationships between overall shifts in the microbiota and a wide range of diseases and conditions. However, existing work has primarily focused on analysis of relatively simple dichotomous or quantitative outcomes, for example, disease status or biomarker levels. Recently, there is also considerable interest in the relationship between the microbiota and censored survival outcomes, such as in clinical trials. How to conduct community-level analysis with censored survival outcomes is unclear, since standard dissimilarity-based tests cannot accommodate censored survival times and no alternative methods exist.

**Methods:**

We develop a new approach, MiRKAT-S, for community-level analysis of microbiome data with censored survival times. MiRKAT-S uses ecologically informative distance metrics, such as the UniFrac distances, to generate matrices of pairwise distances between individuals’ taxonomic profiles. The distance matrices are transformed into kernel (similarity) matrices, which are used to compare similarity in the microbiota to similarity in survival times between individuals.

**Results:**

Simulation studies using synthetic microbial communities demonstrate correct control of type I error and adequate power. We also apply MiRKAT-S to examine the relationship between the gut microbiota and survival after allogeneic blood or bone marrow transplant.

**Conclusions:**

We present MiRKAT-S, a method that facilitates community-level analysis of the association between the microbiota and survival outcomes and therefore provides a new approach to analysis of microbiome data arising from clinical trials.

**Electronic supplementary material:**

The online version of this article (doi:10.1186/s40168-017-0239-9) contains supplementary material, which is available to authorized users.

## Background

The human microbiota, or the collection of microorganisms that inhabits the human body, plays an important role in many areas of health and disease. The development of next-generation sequencing technologies allows culture-free profiling of entire microbial communities, often by sequencing the 16S ribosomal RNA (rRNA) gene [1–3]. Similar 16S sequences are clustered into operational taxonomic units (OTUs); when clustered at the level of 97% similarity, these OTUs represent bacterial species [4]. Using these methods, the human microbiome has been studied at a variety of body sites including the gut [5], skin [6], and respiratory tract [7] and has been associated with many health conditions, such as inflammatory bowel disease, diabetes, psoriasis, and chronic obstructive pulmonary disease [8–10].

Associations between the human microbiota and health outcomes can be assessed by comparing individual OTU abundances or overall diversity metrics between samples or conditions [5, 11]. However, since taxonomic profiles are sparse and high-dimensional—hundreds to thousands of unique OTUs may be identified, many of which are present in only a subset of samples—comparisons on the level of individual OTUs may have low power. An alternative to OTU-level analysis is to compare the microbiota at the community level, i.e., to compare overall taxonomic profiles between individuals [12–14]. This class of analyses is often performed by computing pairwise distances between communities (samples), where the distance metrics are ecologically relevant and may incorporate phylogenetic structure. The matrix of pairwise distances is summarized by its top principal coordinates for visualization, and distance-based multivariate methods coupled with permutation are used to determine if dissimilarity is related to an outcome. Community-level analyses may provide power gains by utilizing phylogenetic information, avoiding the multiple testing problem, and aggregating modest effects across multiple taxa [15].

Recently, as an alternative to distance-based approaches that use permutation analysis, Zhao et al. [14] proposed the microbiome regression-based kernel association test (MiRKAT). MiRKAT uses a kernel machine framework with a variety of ecologically informative kernels to test for associations between the human microbiota and either continuous or binary outcomes. Intuitively, MiRKAT compares similarity in taxonomic profiles between communities (where similarity is measured via a kernel, which can be obtained by transforming relevant distance matrices) to similarity in outcome measures. *p* values are obtained analytically using a variance-component score test. MiRKAT is equivalent to distance-based analysis but has the added advantages of flexible modeling of the relationship between the microbiota and outcome measures, natural incorporation of covariates, and efficient computation of *p* values.

A limitation of existing community-level analysis approaches is that they cannot accommodate censored survival outcomes. However, such outcomes are of tremendous interest as microbiome profiling studies move into the clinical arena. For example, the lung microbiota has been related to progression of idiopathic pulmonary fibrosis [16] and the gut microbiota to overall survival after allogeneic blood and bone marrow transplant [5]. Additional OTU-level studies with survival outcomes have shown associations between the intestinal microbiota and development of atopic dermatitis [17] and allergic rhinitis [18] in children.

To address this critical gap in the literature, in this paper, we propose a test for association between the microbiota and censored survival outcomes (MiRKAT-S), accounting for covariates and potential confounders. We perform a distance-based analysis using the kernel machine Cox regression framework, encoding taxonomic profiles into kernel matrices via a transformation of distance metrics appropriate for microbial communities. This allows the analysis to take into account phylogenetic information and other features specific to biological communities. To formally test the association between the microbiota, as encoded in the kernel matrix, and censored survival times, we use a variance-component score test. However, when applied to microbial community profiles summarized by common kernels, the usual test statistic with *p* values calculated by resampling procedures is highly conservative [19, 20]. We therefore implement a small sample correction that provides proper control of type I error while maintaining adequate power, and we calculate *p* values analytically rather than by resampling. We demonstrate the performance of MiRKAT-S using real and simulated data summarized by a variety of kernels commonly used in microbial ecology.

This work represents the translation of existing methods in genetic studies with survival outcomes to applications in microbiome research. The first major contribution of this work is to allow survival outcomes in the kernel machine regression framework with kernels that appropriately encode microbiome data. Our small sample correction method provides proper control of type I error and improved power when using microbiota-appropriate kernels, whereas the kernel machine regression-based test as implemented for genetic studies has almost no power to detect relationships between microbial taxonomic profiles and survival. Secondly, the ability to perform the test using a variety of kernels provides robustness to the nature of the true association between the microbiota and survival. Therefore, although MiRKAT-S is technically similar to previous kernel machine regression methods, it enables microbiome analyses that are not possible using existing methods.

## Methods

To associate the microbiota at the community level and censored survival times, we will relate censored survival times to taxonomic profiles using a flexible non-parametric modeling framework. We will assess significance via a variance-component score test which acknowledges the modest sample sizes of the most taxonomic profiling studies. In this section, we first describe the modeling framework, followed by the testing strategy and technical advances necessary to ensure proper control of type I error in this framework. Finally, we describe simulations encompassing a variety of true relationships between the microbiota and survival time.

### Model specification

Suppose that for each of *n* subjects, we observe the microbial taxonomic profile, encoded by a *q*-vector of OTU counts ***Z***_*i*_, and a *p*-vector of other covariates ***X***_*i*_. Let *T*_*i*_ be the survival time and *C*_*i*_ the censoring time for the *i*th subject. We observe the bivariate vector (*Δ*_*i*_, *U*_*i*_), where *U*_*i*_=min(*T*_*i*_,*C*_*i*_) is the observed time and *Δ*_*i*_=*I*(*T*_*i*_≤*C*_*i*_) is the event indicator for subject *i*. We wish to test whether taxonomic profiles ***Z*** are associated with survival time, adjusting for covariates ***X***.

The most commonly used model for censored survival times is the Cox proportional hazards model [21] due to its flexibility and relative robustness. Therefore, to relate *T* and (***X***,***Z***), we propose to use the kernel machine Cox proportional hazards model [19, 20], so that

1 $$\begin{array}{@{}rcl@{}} \lambda(t; \boldsymbol{X}, \boldsymbol{Z}) &=& \lambda_{0}(t) \text{exp} \left[ \boldsymbol{X}\boldsymbol{\beta} + f(\boldsymbol{Z}) \right] \end{array} $$

where *λ*_0_(*t*) is the baseline hazard function. In the kernel machine regression framework, *f*(·) is generated by a positive definite kernel function *K*(·,·), that is, *f*(·) lies in the reproducing kernel Hilbert space $\mathcal {H}_{K}$. Under the representer theorem [22], $f(\boldsymbol {Z}_{i}) = \sum _{i^{\prime }=1}^{n} \alpha _{i^{\prime }} K(\boldsymbol {Z}_{i}, \boldsymbol {Z}_{i^{\prime }})$ for some *α*_1_,…,*α*_*n*_. Choosing different kernel functions *K*(·,·) allows specification of a wide variety of models. For example, the kernel function *f*(***Z***_*i*_)=***Z*** *i*′***γ***, corresponding to the linear kernel $\phantom {\dot {i}\!}K(\boldsymbol {Z}_{i}, \boldsymbol {Z}_{i^{\prime }}) = \boldsymbol {Z}_{i} \boldsymbol {Z}_{i^{\prime }}^{\prime }$, is used to specify a linear model. Kernels are similarity matrices, so each element *K*_*j*,*k*_=*K*(***Z***_*j*_,***Z***_*k*_) represents the pairwise similarity between samples *j* and *k*. Because we use a score test, which depends only on the null model, any kernel will result in a valid test; however, the choice of kernel does affect the power of the test.

To specify relevant models for microbial profiles, we use kernel functions that encode the similarity between the microbiota for two samples via a transformation of pairwise distance metrics. There are many commonly used ecological distance metrics, each with different features and strengths. For example, the UniFrac [23] and generalized UniFrac [24] distances take into account the organization of OTUs into phylogenetic trees, thereby gaining power when clusters of taxa are associated with the outcome. Other distances, such as the Bray-Curtis dissimilarity [25], look at the presence and relative abundance of each OTU regardless of phylogenetic structure. These and other commonly used distance metrics can be used to create distance matrices ***D***, where each element *d*_*ij*_ is a pairwise distance between the taxonomic profiles of two samples. The distance matrices are then transformed to kernels, or similarity matrices, via

$$\boldsymbol{K} = -\frac{1}{2} \left(\boldsymbol{I} - \frac{\boldsymbol{1} \boldsymbol{1}^{\prime}}{n}\right) \boldsymbol{D}^{2} \left(\boldsymbol{I} - \frac{\boldsymbol{1} \boldsymbol{1}^{\prime}}{n}\right) $$

as described in [14]. Here, ***I*** is the identity matrix and ***1*** is an *n*-vector of ones. To ensure that ***K*** is positive semi-definite, we replace negative eigenvalues with zero. That is, we perform an eigenvalue decomposition ***K***=***U*** ***Λ*** ***U***, where ***Λ***=diag(*λ*_1_,…,*λ*_*n*_), and then reconstruct the kernel matrix using the non-negative eigenvalues ***Λ*** ^∗^=diag(max(*λ*_1_,0),…,max(*λ*_*n*_,0)) so that ***K***=***U*** ***Λ*** ^∗^ ***U***.

### Score test

Testing whether taxonomic profiles are associated with the outcome in the kernel machine Cox model corresponds to testing the hypothesis *H*_0_:*f*(***Z***)=***K*** ***α***=0. When the model is re-expressed using kernels as

$$\lambda(t; \boldsymbol{X}, \boldsymbol{Z}) = \lambda_{0}(t) \text{exp}\left[ \boldsymbol{X} \boldsymbol{\beta} + \boldsymbol{K} \boldsymbol{\alpha} \right] $$

where ***K***={*K*(***Z***_*i*_,***Z***_*j*_)}_(*i*,*j*)_, we can estimate (***β***,***α***) by maximizing the penalized log partial likelihood function

$${} \begin{aligned} \text{log}&(PL)\\ &= \sum_{i=1}^{n}\! \int_{0}^{\infty} \text{log}\! \left[ \frac{e^{\boldsymbol{\beta}^{\prime} \boldsymbol{X}_{i} + \boldsymbol{\alpha}^{\prime} \boldsymbol{K}_{i}}}{\sum_{j=1}^{n} Y_{j}(s) e^{\boldsymbol{\beta}^{\prime} \boldsymbol{X}_{j} + \boldsymbol{\alpha}^{\prime} \boldsymbol{K}_{j}}} \right]\! dN_{i}(s) - \frac{c}{2} \boldsymbol{\alpha}^{\prime} \boldsymbol{K} \boldsymbol{\alpha} \end{aligned} $$

where *N*_*i*_(*s*)=*I*(*U*_*i*_≤*s*)*Δ*_*i*_, *c*≥0 is the penalty parameter, and *Y*_*j*_(*s*)=*I*(*U*_*j*_≥*s*) is an indicator that subject *j* is at risk at time *s*. An important relationship between kernel regression and linear mixed models has been described for non-censored outcomes [26]; a similar relationship holds in the Cox model, as discussed in [20]. Therefore, solving the penalized log partial likelihood above is equivalent to fitting the random effects Cox model

$$\lambda(t; \boldsymbol{X}, \boldsymbol{Z}) = \lambda_{0}(t) \text{exp}\left[ \boldsymbol{X}\boldsymbol{\beta} + \boldsymbol{h} \right] $$

where ***h***=(*h*_1_,…,*h*_*n*_) are random effects with mean 0 and variance *τ* ***K***. Then, testing *H*_0_:*f*(***Z***)=***K*** ***α***=0 is equivalent to testing *H*_0_:*τ*=0. This hypothesis can be tested using a variance-component score test. Since a score test only requires fitting the null model *λ*(*t*;***X***,***Z***)=*λ*_0_(*t*)exp[***X*** ***β***], we do not need to estimate *f*(***Z***), so the test is valid even if a poor kernel is chosen. However, choosing a kernel that accurately reflects the true relationship between the microbiota and survival time will provide higher power. Two factors determine how well the kernel reflects the true relationship: first, whether the abundance of the associated taxa matters (versus presence or absence), and second, whether the OTUs related to the outcome are clustered on a phylogenetic tree. For example, since the weighted UniFrac distance encodes both taxon abundance and phylogenetic information, a test based on the weighted UniFrac distance will have higher power when the true association is between the outcome and the abundance of a cluster of OTUs on a phylogenetic tree than when the true association is with the abundance of randomly selected OTUs (of similar frequencies) or with the presence or absence of a set of OTUs.

The variance-component score statistic is

$$Q = \hat{\boldsymbol{M}}^{\prime} \boldsymbol{K} \hat{\boldsymbol{M}} $$

where $\hat {\boldsymbol {M}} = (\hat {M_{1}}, \ldots, \hat {M_{n}})$ is the vector of estimated martingale residuals under the null model, i.e., $\hat {M_{i}} = \Delta _{i} - \int _{0}^{\infty } Y_{i}(t) e^{\hat {\beta }^{\prime } \boldsymbol {X}_{i}} d\hat {\Lambda }_{0}(t)$ [19, 20]. Here, $\hat {\Lambda }_{0}(u) = \sum _{i=1}^{n} \Delta _{i} I(U_{i} \leq u) / \hat {S}_{0} (U_{i})$ is Breslows estimator of the baseline hazard function $\Lambda _{0}(u) = \int _{0}^{u} \lambda _{0}(t) dt$ under the null model and $\hat {S}_{0}(t) = \sum _{i=1}^{n} Y_{i}(t) e^{\hat {\beta }^{\prime } \boldsymbol {X}_{i}}$ is the estimator for the baseline survival function.

*Q* asymptotically follows a mixture of chi-square distributions under the null model. The distribution has been derived for a linear kernel [27] but can be written in general form: by the central limit theorem,

$$\boldsymbol{K}^{1/2} \boldsymbol{M} \sim \mathcal{N}\left(0, P_{0}^{1/2} \boldsymbol{K} P_{0}^{1/2}\right) $$

where *P*_0_=*V*−*V* *X*(*X* ^′^ *V* *X*)^−1^ *X* ^′^ *V* with $ V = \text {diag}\left (\int _{0}^{\infty } Y_{i}(t) e^{\hat {\beta }^{\prime } \boldsymbol {X}_{i}} d\hat {\Lambda }_{0}(t) - w_{i}(\beta, t_{i})^{2}\right)$ and $w_{i}(\beta, t) = e^{\hat {\beta }^{\prime } \boldsymbol {X}_{i}}/\hat {S}_{0}(t)$. Therefore,

$$Q \sim \sum_{i=1}^{n} \tilde{\lambda}_{i} \chi^{2}_{1,i} $$

where $\left (\tilde {\lambda }_{1},\ldots,\tilde {\lambda }_{n}\right)$ are the eigenvalues of $P_{0}^{1/2} \boldsymbol {K} P_{0}^{1/2}$ and $\chi ^{2}_{1,i}$ are independent $\chi ^{2}_{1}$ random variables. Note that ***K*** need not be full rank for this distribution to hold, since *λ*_*i*_=0 for the terms associated with the singular components of ***K***, so those components of the distribution will have weight zero [28].

Thus far, we have assumed that there are no tied survival times. In practice, tied survival times are fairly common due to coarse time measurements resulting from specific visit schedules or study follow-up dates. We use the Efron approximation to accommodate tied survival times [29]. This approximation performs well even with relatively small sample sizes or a high proportion of ties [30].

### Small sample correction

The test outlined above is highly conservative for modest sample sizes and complicated kernels, such as kernels commonly used for the microbiota (see Additional file 1: Table S1). We therefore propose an approximate test using a modified score statistic that accounts for overdispersion. Analogous small-sample corrections have been proposed for quantitative and binary traits [31]. Specifically, we propose the modified score statistic

$$Q^{*} = \frac{\hat{\boldsymbol{M}}^{\prime} \boldsymbol{K} \hat{\boldsymbol{M}}}{\hat{\boldsymbol{M}}^{\prime} \hat{\boldsymbol{M}}}. $$

To derive the distribution of this statistic, we need the covariance of the residuals $\hat {M}$. We use a diagonal small-sample approximation to $\text {Cov}(\hat {M})$ that is motivated by the corresponding weighted linear model at convergence. This approximation is justified both in existing literature (e.g., [32, 33]) and through empirical evidence, namely the rapid convergence of the iteratively reweighted least squares (IRLS) algorithm using this weight matrix to the correct coefficients and the proper empirical type 1 error of our method.

Specifically, the fitted kernel machine Cox model (Eq. 1) is equivalent to a weighted linear model at convergence with weight matrix estimated using an iteratively reweighted least squares (IRLS) algorithm, as described in Appendix 1. We use a diagonal approximation for both the covariance of the residuals $\hat {M}$ and the weight matrix *W* for IRLS. Several versions of *W* have been used for weighted partial least squares in the literature (e.g., [34] and [32]); we use an intermediate version that is diagonal as in [32] and [33] but whose elements are defined by the negative Hessian with respect to ***β*** as in [34].

To express this mathematically, let $\tilde {y} = X\beta + {W}^{-1}\hat {\boldsymbol {M}}$ be the working response. Again, although the covariance matrix of the residuals $\hat {\boldsymbol {M}}$ is non-diagonal, we approximate $\text {Cov}(\hat {M})$ using a diagonal form proportional to the weight matrix *W*. Then, by defining $\tilde {y}^{*} = W^{1/2}\tilde {y}$, *X* ^∗^=*W* ^1/2^ *X*, and *ε* ^∗^=*W* ^1/2^ *ε*, the weighted linear model can be written as

$$\tilde{y}^{*} = X^{*}\beta + \epsilon^{*}, \epsilon^{*} \sim \mathcal{N}(0, \sigma^{2} I) $$

with projection matrix $P_{0}^{*} = I - X^{*}(X^{*^{\prime }}X^{*})^{-1}X^{*^{\prime }}$. This model can be reframed using $\tilde {y}^{*} = W^{1/2}\tilde {y}$, *X* ^∗^=*W* ^1/2^ *X*, and *ε* ^∗^=*W* ^1/2^ *ε* and fit as a weighted linear model. Then, at convergence, Var(*ε* ^∗^) = *W* ^1/2^Var(*ε*) *W* ^1/2^ = *W* ^1/2^ *σ* ^2^ *W* ^−1^ *W* ^1/2^=*σ* ^2^ ***I***.

Based on this, the distribution of *Q* ^∗^ satisfies

2 $$\begin{array}{@{}rcl@{}} P(Q^{*} > q) &=& P(\hat{\boldsymbol{M}}^{\prime} \boldsymbol{K} \hat{\boldsymbol{M}} - \hat{\boldsymbol{M}}^{\prime} q \hat{\boldsymbol{M}} > 0) \\ &=& P((P_{0}^{*} \boldsymbol{M})^{\prime} (\boldsymbol{K} - q\boldsymbol{I}) (P_{0}^{*}\boldsymbol{M}) > 0) \\ &=&P(\epsilon^{\prime} P_{0}^{1/2} P_{0}^{*} (\boldsymbol{K}-q\boldsymbol{I}) P_{0}^{*} P_{0}^{1/2} \epsilon > 0) \end{array} $$

where $\epsilon \sim \mathcal {N}(0,\boldsymbol {I})$. For the second equality, it is fairly straightforward to show that $\hat {\boldsymbol {M}} = P_{0}^{*} \boldsymbol {M}$; this derivation is included in Appendix 2. The third equality uses the distribution of ***M*** stated in the previous section. Then, under the null hypothesis,

$$Q^{*} \sim \sum_{i=1}^{n} \lambda_{i}^{*} \chi^{2}_{1,i} $$

where $(\lambda _{1}^{*}, \ldots, \lambda _{n}^{*})$ are the eigenvalues of $P_{0}^{1/2} P_{0}^{*} (\boldsymbol {K}-q\boldsymbol {I}) P_{0}^{*} P_{0}^{1/2}$ and, as before, $\chi ^{2}_{1,i}$ are independent $\chi ^{2}_{1}$ random variables. *p* values can be calculated efficiently using Davies’ exact method [35]. For very small samples (e.g., *n*≤50), Davies *p* values may be anticonservative and permutation *p* values may be used instead.

### Simulation scenarios

We carried out simulation studies in a range of settings to confirm that MiRKAT-S properly controls type I error and to assess its power using a variety of kernels. Microbiome OTU counts were generated using the same approach as [14]. Specifically, for each individual, we simulated OTU counts from a Dirichlet-multinomial distribution with dispersion parameters and proportions estimated from Charlson et al.’s real upper respiratory tract microbiome dataset, in which 856 OTUs were measured on each of 60 individuals [7]. The data for each simulated individual consists of 1000 total OTU counts distributed among the 856 OTUs of Charlson et al. We also simulated two covariates for each individual, *X*_1*i*_ and *X*_2*i*_, from a standard normal and a Bernoulli (0.5) distribution independently of taxonomic profiles. We considered sample sizes ranging from *n*=25 to *n*=500 individuals. For all simulation scenarios, we generated datasets with approximately 25 and 50% censoring. Four simulation settings were considered, varying (1) whether OTU abundance or the presence/absence of OTUs was associated with the outcome and (2) whether phylogenetically clustered or unclustered OTUs were associated with the outcome.

In setting 1, the abundances of OTUs in one cluster on a phylogenetic tree were associated with survival time. We partitioned all of the OTUs into 20 clusters using the partitioning-around-medioids algorithm based on the cophenetic distances of OTUs in the phylogenetic tree. The abundance of clusters ranged from 0.05 to 19.7% of all OTU reads. We selected an abundant cluster, containing 19.7% of all reads, and a rare cluster, containing 0.9% of all reads, to be associated with exponentially distributed survival times through the model

3 $$\begin{array}{@{}rcl@{}} T_{i} &=& \frac{-\text{log}(U_{i})}{\lambda \text{exp}\left(X_{i}^{\prime} \beta + \gamma \text{scale}\left(\sum_{j \in \mathcal{A}} Z_{ij} \right) \right)} \end{array} $$

where *γ* is the true effect size for the cluster, *λ* is the scale parameter, *U*_*i*_∼Uniform(0,1), $\mathcal {A}$ is the set of indices of OTUs in the selected cluster, and the “scale” function standardizes the total OTU abundance in the cluster to have mean 0 and standard deviation 1:

$$\text{scale}\left(\sum_{j\in \mathcal{A}} Z_{ij} \right) = \frac{\sum_{j\in \mathcal{A}} Z_{ij} - \frac{1}{n} \sum_{i} \left(\sum_{j \in \mathcal{A}} Z_{ij}\right)}{SD_{i} (\left(\sum_{j\in\mathcal{A}} Z_{ij}\right)}. $$

Censoring times are simulated independently as *C*_*i*_∼Exp(*μ*), and *λ* and *μ* are selected to give approximately 25% or approximately 50% censoring.

In setting 2, the ten most abundant OTUs overall, accounting for 31.5% of all OTU reads, were associated with survival time regardless of cluster membership. In this setting, we simulated survival times as

4 $$\begin{array}{@{}rcl@{}} T_{i} &=& \frac{-\text{log}(U_{i})}{\lambda \text{exp}\left(X_{i}^{\prime} \beta + \gamma \text{scale}\left(\sum_{j \in \mathcal{A}} \frac{Z_{i(j)}}{\bar{Z}_{(j)}} \right) \right)} \end{array} $$

where $\bar {Z}_{(j)}$ is the average across samples of the counts for the *j*th OTU. This limits the ability of a single OTU to dominate the communal effect of the microbiota. Setting 2 is comparable to setting 1 when the associated cluster is common, since in both cases the abundance of common OTUs is associated with survival times, but it lacks setting 1’s close phylogenetic relationship between associated OTUs.

In setting 3, the presence or absence of each OTU in a cluster was associated with survival time. OTUs were clustered as in setting 1, but in this case, were associated with survival time via the model

5 $$\begin{array}{@{}rcl@{}} T_{i} &=& \frac{-\text{log}(U_{i}) }{ \lambda \text{exp}\left(X_{i}^{\prime} \beta + \gamma \text{scale}\left(\sum_{j \in \mathcal{A}} I(Z_{ij}>0) \right) \right)} \end{array} $$

As in setting 1, we simulated situations where an abundant cluster, containing 19.7% of all reads, was associated with the outcome and where a rare cluster, containing 0.9% of all reads, was associated with the outcome.

Finally, in setting 4, the presence or absence of 40 randomly selected OTUs was associated with the survival time. This mimics the size of an average cluster, since the mean number of OTUs assigned to a cluster was 42.8, with cluster sizes ranging from 3 to 118 OTUs. Since the majority of OTUs are rare, the overall number of OTU reads associated with the outcome is low in this setting. The model for *T*_*i*_ was the same as in setting 3. Setting 4 is comparable to setting 3 when the associated cluster is rare: in both cases, the presence or absence of rare OTUs is associated with survival times. However, it lacks setting 3’s close phylogenetic relationship between associated OTUs.

In all simulation settings, we considered the weighted (*K*_w_) and unweighted (*K*_u_) UniFrac kernels, the Bray-Curtis kernel (*K*_BC_), and the generalized UniFrac kernel with *α*=0.5 (*K*_0.5_). These kernels are expected to have high power in different simulation settings. All of the UniFrac kernels take phylogenetic information into account. The unweighted UniFrac kernel does not account for OTU abundance, whereas the weighted UniFrac kernel does; the generalized UniFrac kernel is intermediate between the weighted and unweighted. The Bray-Curtis kernel does not account for phylogenetic structure or overall abundance of an OTU but does compare both presence/absence and relative abundance between samples of each OTU. Each kernel will have highest power when its measure of distance (and therefore similarity) accurately reflects the true relationship between the microbiome and the outcome.

For each simulation setting, sample size *n*, and censoring proportion, and using each kernel, we applied the test described above to test for associations between OTU counts and survival time. We used 5000 simulations with *γ*=0 to estimate the empirical type I error rate with a nominal significance level of 0.05 and estimated empirical power across a range of *γ* values using 1000 simulations.

We also compare MiRKAT-S to two alternative approaches sometimes used for community-level analysis. First, we performed OTU-level tests of all OTUs. For each of the 856 OTUs in the dataset, we ran a marginal Cox regression model. The minimum *p* value from the 856 marginal models was compared to the null distribution to produce an overall *p* value for any association of the microbiota with survival times. In practice, the null distribution would be generated for an individual study using permutation; however, in the interest of computational efficiency, we generated this distribution using the minimum *p* values from 5000 simulations where survival times were not associated with the microbiota. Second, we performed principal coordinates analysis (PCoA) on a relevant distance matrix (see, e.g., [16]). Since it is not clear how to make PCoA plots with censored time-to-event outcomes, we followed PCoA by Cox proportional hazards regression. Specifically, we generated the UniFrac and Bray-Curtis distance matrices as above, then took the top two principal coordinates as our covariates for a Cox regression analysis. We tested the two principal coordinates jointly by using a chi-square test to compare nested models with and without the microbiota-related predictors. The covariates *X*_1_ and *X*_2_ were included in all models exactly as in the MiRKAT-S simulations.

## Results

### Power and type I error in simulated datasets

Empirical type I error rates with 25% censoring are reported in Table 1. Note that Eqs. 3, 4, and 5 are identical when *γ*=0 (i.e., there is no true association between the microbiota and survival time), so settings 1–4 all have the same type I error. From the table, we see that MiRKAT-S is valid for all kernels and sample sizes of at least 100 individuals. For comparison, empirical estimates of type I error without the small-sample correction are reported in (Additional file 1: Table S1), demonstrating the uncorrected test is highly conservative. To further describe the behavior of *p* values based on the uncorrected test statistic, we have generated Q-Q plots (see Additional file 1: Figure S1). These plots demonstrate that *p* values based on the corrected statistic do not deviate significantly from the theoretical distribution, whereas *p* values based on the uncorrected statistic are far from the theoretical distribution. They also show that, more specifically, *p* values based on the uncorrected statistic tend to be less extreme than they should be: *p* values that are truly smaller than 0.2 tend to be overestimated, whereas *p* values larger than 0.2 tend to be underestimated. All type I error results are similar with 50% censoring (see Additional file 1: Table S2). For sample sizes smaller than 100, the size of MiRKAT-S is close to correct, though it may be slightly anticonservative. Empirical type I errors for small sample sizes are reported in Table 2. If the sample size is smaller than *n*=50 and the *p* value from MiRKAT-S is borderline, it may be preferable to report *p* values obtained using permutation.

**Table 1:** Empirical type I errors for *n*=100, 200, or 500

Number	*K*_w_	*K*_u_	*K*_0.5_	*K*_BC_
100	0.0544	0.0540	0.0530	0.0542
200	0.0494	0.0480	0.0470	0.0462
500	0.0506	0.0478	0.0536	0.0442

Empirical type I errors for sample sizes *n*=100, 200, and 500 with approximately 25% censoring. Results are based on 5000 simulated datasets. *K*_w_, *K*_u_, *K*_BC_, and *K*_0.5_ represent results for the weighted UniFrac kernel, unweighted UniFrac kernel, Bray-Curtis kernel, and generalized UniFrac kernel with *α*=0.5, respectively

**Table 2:** Empirical type I errors for *n*<100

Number	Method	*K*_w_	*K*_u_	*K*_0.5_	*K*_*BC*_
25	MiRKAT-S	0.054	0.055	0.055	0.056
	Permutation	0.046	0.048	0.046	0.049
50	MiRKAT-S	0.045	0.058	0.051	0.051
	Permutation	0.041	0.052	0.045	0.045
75	MiRKAT-S	0.054	0.058	0.051	0.053
	Permutation	0.051	0.053	0.048	0.049

Empirical type I errors for small sample sizes (*n*<100) with approximately 25% censoring. Results are based on 5000 simulated datasets, and permutation *p* values were obtained using 1000 permutations. *K*_w_, *K*_u_, *K*_0.5_, and *K*_BC_ represent results for the weighted UniFrac kernel, unweighted UniFrac kernel, Bray-Curtis kernel, and generalized UniFrac kernel with *α*=0.5, respectively

To interpret the simulation results evaluating the power of the test, recall that two aspects of the relationship between the microbiome and survival are important for understanding which kernel will provide the highest power: the relationship between associated OTUs (whether or not they cluster on a phylogenetic tree) and the importance of taxon abundance (whether OTU count or presence/absence matters). All of the UniFrac distances account for phylogeny, while the Bray-Curtis dissimilarity does not. The weighted UniFrac distance and Bray-Curtis dissimilarity both utilize taxon abundance (OTU counts), whereas the unweighted UniFrac distance only incorporates presence/absence of taxa, and the generalized UniFrac distance is a compromise between the weighted and unweighted UniFrac distances.

**Fig. 1:**
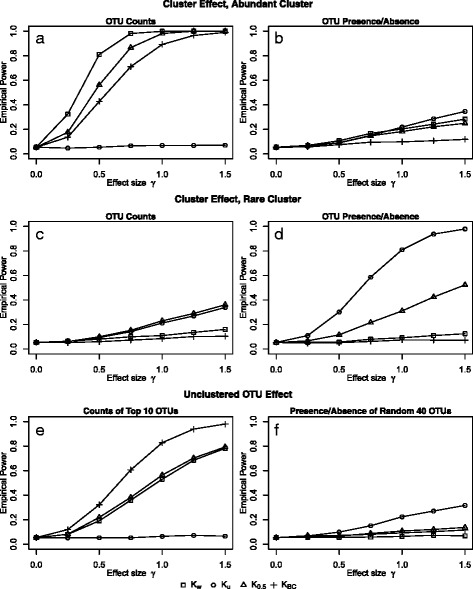
Empirical power. Empirical power was evaluated in all simulation settings, using a sample size of *n*=100 and 25% censoring. *K*_w_, *K*_u_, *K*_BC_, and *K*_0.5_ represent results for the weighted UniFrac kernel, unweighted UniFrac kernel, Bray-Curtis kernel, and generalized UniFrac kernel with *α*=0.5, respectively. *γ* is the true effect size for the associated cluster or OTUs. **a** Setting 1; survival is associated with OTU counts in a common cluster containing 19.7% of all reads. **b** Setting 3; survival is associated with the presence or absence of each taxon in a common cluster. **c** Setting 1; survival is associated with OTU counts in a rare cluster containing 0.9% of all reads. **d** Setting 3; survival is associated with the presence or absence of each taxon in a rare cluster. **e** Setting 2; survival is associated with the ten most common OTUs, regardless of cluster membership. **f** Setting 4; survival is associated with 40 OTUs selected at random, regardless of cluster membership

Figure 1 shows the estimated power under all simulation settings. As expected, in all settings, power increases with increasing true effect size (*γ*). We first consider settings 1 and 3, in which a cluster of OTUs is associated with the outcome. When the OTU counts of an abundant cluster are associated with survival times (panel A), the weighted UniFrac kernel and the generalized UniFrac kernel with *α*=0.5 provide the highest power, since the corresponding distance metrics take both abundance and phylogeny into consideration. Since the associated cluster is common, nearly all individuals have at least one read for each OTU in the cluster, so individuals cannot be distinguished based on OTU presence/absence. Therefore, in this setting, the unweighted UniFrac kernel has almost no power to detect the association. In contrast, when OTU presence/absence in a rare cluster is associated with survival time (panel D), the unweighted UniFrac kernel has highest power, since this distance metric is based on the presence and absence of OTUs. Here, the weighted UniFrac kernel has very low power because OTU counts of a rare cluster do not vary much between individuals. When the OTU counts of a rare cluster are associated with survival time (panel C) or when the presence or absence of an abundant cluster is associated with survival time (panel B), the OTU effect is small and similar across most individuals. Therefore, in these settings, no kernel provided power comparable to settings with larger effect sizes.

The power under settings 2 and 4, in which unclustered OTUs are associated with the outcome, is reported in panels e and f of Fig. 1. When the OTU counts of the ten most common OTUs are associated with survival time (panel e), the Bray-Curtis kernel has highest power, followed by the weighted UniFrac kernel and generalized UniFrac kernel with *α*=0.5. Since the Bray-Curtis dissimilarity metric does not incorporate phylogenetic information, this distance is designed for unclustered rather than clustered OTUs. However, since it takes abundance into account, the Bray-Curtis kernel performs better when OTU counts are associated with survival (e.g., panel a) rather than OTU presence/absence (e.g., panel d) and when the associated cluster is at least moderately abundant. When the presence or absence of a random 40 OTUs were associated with survival time (panel f), no kernel had high power, but the unweighted UniFrac kernel had higher power than any other tested kernel. The low power is likely due to the rarity of most randomly selected clusters and inability to gain power by utilizing phylogenetic information.

Kernel choice has a strong effect on the power of the test, and different kernels are optimal depending on the nature of the true relationship between the microbiota and survival time. In practice, a kernel representing relationships of particular scientific interest could be selected. For example, if a healthy microbiome at a certain body site has relatively few dominant taxa at high frequencies, and changes in the relative abundance of these taxa is hypothesized to be associated with the time to a disease outcome or death, choosing a kernel that accounts for taxon abundance will have the highest power to detect the hypothesized changes. If there is no specific hypothesized relationship, multiple kernels can be tested and then the resulting *p* values adjusted for multiple comparisons. Testing the four kernels discussed here is a reasonable starting point, and the limited number of tests reduces the power loss due to adjusting for multiple comparisons. In these simulations, if the analysis is performed using all four kernels included in Fig. 1 (*K*_u_,*K*_0.5_,*K*_w_,*K*_BC_) and then the minimum *p* value after an FDR adjustment is used for testing, the power does not quite match the best kernel but is comparable to or better than the remaining three kernels (see Additional file 1: Figure S2).

We also compared the power of MiRKAT-S to two approaches used in current practice: performing a marginal, or OTU-level, analysis for all OTUs and including the top principal coordinates of the distance matrix as the covariates of interest in a regression model (see Additional file 1: Figure S2). We find that for most simulation settings, MiRKAT-S has substantially better power than the marginal analysis or PCoA, and in the remainder, the power is comparable between methods. In particular, the marginal analysis has power to detect an association between counts of OTUs in a cluster and survival times, but virtually no power to detect associations between presence/absence of clustered OTUs and survival or associations involving unclustered OTUs. The power of the marginal test for detecting an association with clustered OTU counts has similar power regardless of how common the cluster is. Therefore, since MiRKAT-S is more powerful for OTU counts of relatively common clusters, the marginal analysis and MiRKAT-S have similar power for rare clusters, and for very large effect sizes in this simulation setting, the marginal analysis outperforms MiRKAT-S slightly (Additional file 1: Figure S2 (panel C)). However, in all other cases, MiRKAT-S is substantially more powerful than the OTU-level analysis. PCoA regression analysis performs similarly to MiRKAT-S for each kernel when OTU counts in a common cluster are associated with survival times (Additional file 1: Figure S2 (panel A)). In most other simulation settings, PCoA matches or approaches the power of MiRKAT-S in only the case of the best kernel. That is, MiRKAT-S is more robust to kernel choice than PCoA. In addition, in settings where clustering information does not matter (Additional file 1: Figure S2 (panels e and f)), PCoA has very low power, while MiRKAT-S has moderate power provided that the associated OTUs are not too rare.

### Analysis of blood and bone marrow transplant data

Acute graft-versus-host disease (aGVHD) is a leading cause of death after allogeneic blood or bone marrow transplantation. There is a suspected relationship between the intestinal microbiome and aGVHD, but previous studies in mice and humans have yielded mixed results about the presence and nature of this relationship. Therefore, Jenq et al. recently studied the association of a particular bacterial species (intestinal *Blautia*) and of intestinal microbiome diversity indices with time to each of aGVHD onset, aGVHD-related mortality, and adverse outcomes unrelated to aGVHD [5].

In the original study, subjects were stratified into two cohorts depending on sequencing platform. The combined dataset used here results from resequencing of the first cohort of patients using the Illumina MiSeq platform; unfortunately, four patients did not have additional DNA available for MiSeq sequencing and were excluded from our analysis. Therefore, 481 stool samples were available for 111 unique subjects, and for each sample, the Illumina MiSeq platform was used to sequence the V4–V5 regions of the 16S rRNA gene. OTUs were generated as described in [5]. Briefly, mothur version 1.34 was used to compile and process sequence data [36], and quality filters were applied as in [37]. This procedure yielded OTU counts for 2436 OTUs. As in the original paper, for each subject, we only included the sample collected closest to 12 days post-transplant in our analysis, and we excluded subjects for whom no samples were collected between 8 and 16 days post-transplant, so that 94 subjects were included. We used QIIME with default settings to align the sequences and generate a rooted phylogenetic tree. The 109 OTUs that failed to be placed on the tree were excluded from our analysis, leaving 2327 OTUs. We performed the test using the unweighted and weighted UniFrac kernels, the generalized UniFrac kernel with *α*=0.5, and the Bray-Curtis kernel, adjusting for age and gender. The outcomes considered were overall survival and time to stage III aGVHD.

The results of applying MiRKAT-S to these data with and without the small-sample correction are reported in Table 3. We do not find a significant association between the microbiota and time until development of stage III aGVHD using any kernel. However, the association between overall survival and the microbiota is significant at *α*=0.05 using the unweighted UniFrac kernel *K*_u_, generalized UniFrac kernel *K*_0.5_, and Bray-Curtis kernel *K*_BC_, but not using the weighted UniFrac kernel *K*_w_ (Table 3). The association remains significant after we adjust for multiple comparisons (multiple kernels) using either the false discovery rate method or the Bonferroni correction. The differences between the corrected and uncorrected *p* values are fairly small. However, they are in the direction we would expect based on simulation results. In particular, we saw that low and high *p* values are less frequent than would be expected for a null distribution of *p* values (see Additional file 1: Figure S1). This is consistent with seeing higher *p* values for the uncorrected statistic in the overall survival case, where the *p* values based on the corrected statistic are fairly small, and conversely, seeing lower *p* values for the uncorrected statistic in the grade III aGVHD case, where the *p* values based on the corrected statistic are large.

**Table 3:** Analysis of gut microbiome after allogeneic transplant

Outcome	Method	*K*_u_	*K*_0.5_	*K*_w_	*K*_BC_
Overall Survival	Uncorrected	0.049	0.008	0.065	0.029
	Corrected	0.046	0.007	0.065	0.022
Grade III aGVHD	Uncorrected	0.496	0.514	0.472	0.849
	Corrected	0.560	0.575	0.518	0.933

*p* values from MiRKAT-S using the weighted (*K*_w_) and unweighted (*K*_u_) UniFrac kernels, the generalized UniFrac kernel with *α*=0.5 (*K*_0.5_), and the Bray-Curtis kernel (*K*_BC_) with outcomes of overall survival and severe (at least grade 3) graft-versus-host disease. “Corrected” indicates the *p* values are based on the modified score statistic with proper type I error; “uncorrected” indicates the *p* values are based on the original score statistic

**Fig. 2:**
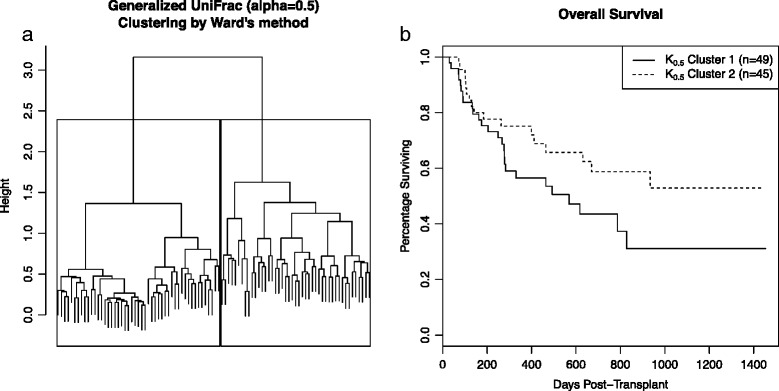
Cluster analysis. **a** Clustering of individuals using Ward’s hierarchical clustering method, based on generalized UniFrac distances with *α*=0.5. **b** Kaplan-Meier curves for the two clusters of individuals with an outcome of overall survival

To visualize this association, we clustered individuals using Ward’s agglomerative hierarchical clustering method [38] based on the generalized UniFrac distance with *α*=0.5. Ward’s method is a generic clustering method that can be used for many data types. Generally speaking, the goal is to divide samples into clusters (groups) that tend to be similar in the ways that we care about; here, clusters should reflect similarity of taxonomic profiles. Operationally, Ward’s method begins by assigning each sample to its own cluster and sequentially merges pairs of clusters that are most similar into larger clusters until all samples are merged into a single cluster. Which clusters to merge is decided by minimizing the increase in the sum of within-cluster squared distances (when Euclidean distances are used, this is the within-cluster variance). Through this process, a hierarchical tree is created. The tree can be cut at different levels to create the desired number of final clusters used for analysis. Although Euclidean distances are often used for Ward’s method, other squared distances (in this case, ecologically relevant metrics such as the UniFrac distances) can be substituted while still using the same form of criterion and algorithm [39]. For our analysis, we used the generalized UniFrac distance to measure dissimilarity between individuals to ensure that clusters are similar with regard to the presence and abundance of taxa, accounting for phylogenetic relationships. We chose to cut the tree to create two clusters; a clear separation into clusters of sizes *n*=45 and *n*=49 can be seen in Fig. 2 a.

Kaplan-Meier curves for overall survival in the two clusters are shown in Fig. 2 b. However, the simple Cox regression *p* value is not significant (*p*=0.09). That is, the similarity between individuals was measured the same way in both analyses. MiRKAT-S yielded a highly significant *p* value for the association of the microbiota with overall survival, whereas the analysis based on clustering individuals gave a non-significant *p* value. This demonstrates that MiRKAT-S has higher power to detect this association than a simple clustering analysis based on the same distance metric.

The highly significant result using MiRKAT-S with *K*_0.5_ may also provide information about the form of the association between the gut microbiota and survival post-transplant. The generalized UniFrac kernel incorporates phylogenetic information and represents a compromise between abundance and presence or absence of OTUs. Therefore, this kernel has highest power to detect relationships between taxonomic profiles and overall survival that occur through moderately rare clusters of OTUs or through a combination of common and rare clusters of OTUs. Accordingly, the high significance of MiRKAT-S using *K*_0.5_ may indicate that one of those settings holds: either moderately rare clusters of OTUs are driving the relationship between the microbiota and overall survival, or multiple clusters of OTUs, some of which are abundant and some of which are rare, are associated with overall survival. However, without further analysis, we cannot determine which OTUs or clusters are associated with survival times in aGVHD patients.

## Discussion

We propose MiRKAT-S for testing the association between the human microbiota and survival outcomes. In the kernel machine Cox model framework, taxonomic profiles are modeled through a kernel function. This allows comparison of microbial community profiles using microbiome-specific distance metrics such as the UniFrac distances or Bray-Curtis dissimilarity. The kernel machine regression framework also allows linear (or, more generally, parametric) adjustment for covariates and potential confounders. We test the significance of the association between the microbiota and survival times using a variance-component score test, and we develop a small-sample correction to account for the modest sample sizes and sparse, high-dimensional data that often result from microbiome studies. In contrast to existing methods that use resampling, *p* values are obtained analytically using the Davies approximation.

Like other distance-based analyses, MiRKAT-S is limited to detecting the presence of an association between the microbiota and survival times. It cannot identify individual taxa that are associated with the outcome and does not provide information about relationships among taxa within a microbial community. MiRKAT-S is therefore designed to be used when the question of interest is whether an entire microbial community is associated with the outcome. Alternative ways to answer this question include testing the association of each OTU individually with the outcome of interest or using a dimension reduction technique such as PCoA and testing the top few principal coordinates. Our simulation studies show that MiRKAT-S has power at least comparable to, and often substantially greater than, either of these methods for community-level association testing. Community-level tests can be used in combination with other methods that identify taxa of interest. These include marginal tests for particular OTUs of interest, identification of OTUs with high loadings from PCA or PCoA, or penalized regression methods that account for the structure and compositional nature of the data.

Our simulation results show that MiRKAT-S correctly controls type I error. However, under conditions of extreme censoring or very small sample sizes, the analytic *p* values provided by MiRKAT-S may be slightly anticonservative. In these cases, obtaining *p* values by permutation may be preferable. Type I error is accurate regardless of the choice of kernel; that is, the test is valid even when a poor choice of kernel is made. The power of the test depends heavily on how well the selected kernel encodes the true relationship between the microbiome and the outcome of interest. For example, when the abundance of an OTU or set of OTUs is related to the outcome, a kernel that encodes abundance information, such as the weighted UniFrac or Bray-Curtis kernel, will have higher power than a kernel that encodes only taxon presence or absence. There are situations in which MiRKAT-S has low power regardless of kernel choice, but any method would have low power in those settings because the true effect size is very small. For example, if the presence or absence of a common OTU is associated with the outcome, nearly all subjects will have the OTU present in the sample, so the association will be very difficult to detect using any method.

If there is no a priori hypothesis about which kernel will best represent relationships of scientific interest, the analysis can be performed using multiple kernels and an overall *p* value can be obtained by permutation or adjustment for multiple comparisons. This analysis approach can provide information not only about the presence of a relationship but also about its form, depending on the distance metrics considered and their relative power for different forms of the true association. That is, if the metric with the lowest *p* value has the highest power to detect associations with abundance of common clusters, that may be the form of the true association. Furthermore, weighted combinations of kernels could be used to simultaneously detect different types of shifts in the microbiota. Specific combinations or kernel weights could either be selected a priori or via a grid search, again using permutation to test overall significance. As the field of microbiome analysis matures and new distance metrics are proposed, our approach will continue to increase in power.

## Conclusions

We present MiRKAT-S, a method for testing the association between the microbiota, assessed on the community level, and survival (time-to-event) outcomes. Similar methods exist for binary and continuous outcomes; however, MiRKAT-S is the first community-level test for microbiome data that allows analysis of censored survival outcomes. Community-level analyses have several benefits: they often provide higher statistical power to detect associations, and they allow investigators to address additional scientific questions, such as whether the entire microbiome is collectively associated with survival time or time to development of a disease. We use the kernel machine regression framework, encoding microbiome data in ecologically relevant kernels. With judicious choice of kernels, the test can detect a wide range of true forms of association, including association of the outcome with OTU presence/absence or abundance and with either phylogenetically clustered or unclustered sets of taxa. Therefore, MiRKAT-S facilitates a robust community-level analysis of the association between the microbiota and censored survival outcomes that is not possible using existing methods.

## Appendix 1

An iteratively reweighted least squares (IRLS) algorithm can be used to fit the linear model at convergence that is equivalent to the Cox PH model of interest. At the *k*th step of the IRLS algorithm, we solve

$$\tilde{y}^{k} = X\beta + \epsilon, \quad \quad \epsilon \sim \mathcal{N}\left(0, \sigma^{2} (\tilde{W}^{k})^{-1}\right) $$

with weight matrix

$$\begin{aligned} \tilde{W}^{k} &= \text{diag} \left(\int_{0}^{\infty} I(T_{i} \geq t) e^{X_{i}^{\prime} \tilde{\beta}^{k}} d\hat{\Lambda}_{0}(t)\right.\\ &\left.\quad- \int_{0}^{\infty} I(T_{i} \geq t) w_{i}(\tilde{\beta}^{k}, t) e^{X_{i}^{\prime} \tilde{\beta}^{k}} d\hat{\Lambda}_{0}(t) \right) \end{aligned} $$

where $w_{i}(t) = \frac {e^{X_{i}^{\top } \beta }}{\sum _{l=1}^{n} Y_{l}(t)e^{X_{l}^{\prime }\beta }}$ and working response

$$\tilde{y} = X\tilde{\beta}^{k-1} + \left(\tilde{W}^{k-1}\right)^{-1}\hat{\boldsymbol{M}}^{k-1}. $$

The corresponding quantities without the superscript *k* refer to the model at convergence. Then, the modified score statistic is equivalent to

$$Q^{*} = \frac{(\tilde{y} - X\tilde{\beta})^{\prime} \tilde{W} \boldsymbol{K} \tilde{W} (\tilde{y} - X\tilde{\beta})}{ \hat{\sigma}^{2} } $$

which is analogous to the linear and logistic cases considered in [31]. Multiplying both sides of the equation by $\tilde {W}^{1/2}$ and defining $\tilde {y}^{*} = W^{1/2}\tilde {y}$, *X* ^∗^=*W* ^1/2^ *X*, and *ε* ^∗^=*W* ^1/2^ *ε*, the model can be expressed as

$$\tilde{y}^{*} = X^{*}\beta + \epsilon^{*}, \epsilon^{*} \sim \mathcal{N}(0, \sigma^{2} I) $$

with projection matrix $P_{0}^{*} = I - X^{*}(X^{*^{\prime }}X^{*})^{-1}X^{*^{\prime }}$.

## Appendix 2

To derive the relationship between $\hat {\boldsymbol {M}}$ and ***M***, recall that

$$\begin{array}{@{}rcl@{}} \hat{z} &=& X\hat{\beta} + W^{-1} \hat{M}  \\ z &=& X\beta + W^{-1}M  \\ \hat{\beta} &=& (X^{\prime}WX)^{-1} X^{\prime}Wz  \end{array} $$

Then, solving the first equation for $\hat {M}$ gives

$$\begin{array}{@{}rcl@{}} \hat{\boldsymbol{M}} &=& W(z-X\hat{\beta}) = W\left[ X\beta + W^{-1}\boldsymbol{M} - X\hat{\beta} \right]  \\ &=& W \left[ I - X(X^{\prime}WX)^{-1} X^{\prime}W \right] z  \\ &=& W \left[ I - X(X^{\prime}WX)^{-1} X^{\prime}W \right] (X\beta + W^{-1}M)  \\ &=& \left[WW^{-1}M - WX(X^{\prime}WX)^{-1} X^{\prime}W W^{-1}M \right]  \\ &=& \left[I - WX(X^{\prime}WX)^{-1}X^{\prime}\right] M  \\ &=& \left[I - X^{*}(X^{*^{\prime}}X^{*})^{-1} X^{*}\right] M = P_{0}^{*} M  \end{array} $$

so that $\hat {M} = P_{0}^{*} M$, as claimed.

## Additional file

Additional file 1 PDF file includes supplemental tables (**Tables S1**–**S3**) and figures (**Figures S1**–**S2**). (PDF 933 kb)
