# Supplementary material for: MiRKAT-S: a community-level test of association between the microbiota and survival times
Source: Microbiome. 2017 Feb 8;5:17. doi: 10.1186/s40168-017-0239-9 (PMC5299808; doi:10.1186/s40168-017-0239-9)
Supplement: Additional file 1 — PDF file includes supplemental tables (Tables S1–S3) and figures (Figures S1–S2). (PDF 933 kb) [file 40168_2017_239_MOESM1_ESM.pdf]

Supplementary Material

MiRKAT-S: A community-level test of association between the microbiota and survival times

Anna Plantinga, Xiang Zhan, Ni Zhao, Jun Chen, Robert R. Jenq, Michael C. Wu

Table S1: Empirical Type I errors when no small sample correction is used, with approximately 25% censoring. Results are based on 5,000 simulated datasets. K_u, $K_{0.5}$, K_w, and K_{BC} represent results for the unweighted UniFrac kernel, generalized UniFrac kernel with $\alpha = 0.5$, weighted UniFrac kernel, and Bray-Curtis kernel, respectively.

n	K_u	$K_{0.5}$	K_w	K_{BC}
100	0.0102	0.0084	0.0286	0.0144
200	0.0162	0.0130	0.0308	0.0188
500	0.0218	0.0248	0.0364	0.0254

Table S2: Empirical Type I errors with approximately 50% censoring. Results are based on 5,000 simulated datasets. K_u, $K_{0.5}$, K_w, and K_{BC} represent results for the unweighted UniFrac kernel, generalized UniFrac kernel with $\alpha = 0.5$, weighted UniFrac kernel, and Bray-Curtis kernel, respectively.

n	K_u	$K_{0.5}$	K_w	K_{BC}
100	0.0546	0.0528	0.0504	0.0512
200	0.0474	0.0494	0.0522	0.0516
500	0.0470	0.0450	0.0478	0.0448

Table S3: Empirical Type I errors for small sample sizes ($n < 100$) with approximately 25% censoring. Results are based on 5,000 simulated datasets and permutation p-values were obtained using 1000 permutations. K_u, $K_{0.5}$, K_w, and K_{BC} represent results for the unweighted UniFrac kernel, generalized UniFrac kernel with $\alpha = 0.5$, weighted UniFrac kernel, and Bray-Curtis kernel, respectively.

n	Method	K_u	$K_{0.5}$	K_w	K_{BC}
25	No Small Sample Correction	0.007	0.007	0.018	0.009
	MiRKAT-S	0.055	0.055	0.054	0.056
	Permutation	0.048	0.046	0.046	0.049
50	No Small Sample Correction	0.006	0.004	0.016	0.005
	MiRKAT-S	0.058	0.051	0.045	0.051
	Permutation	0.052	0.045	0.041	0.045
75	No Small Sample Correction	0.009	0.008	0.029	0.012
	MiRKAT-S	0.058	0.051	0.054	0.053
	Permutation	0.053	0.048	0.051	0.049

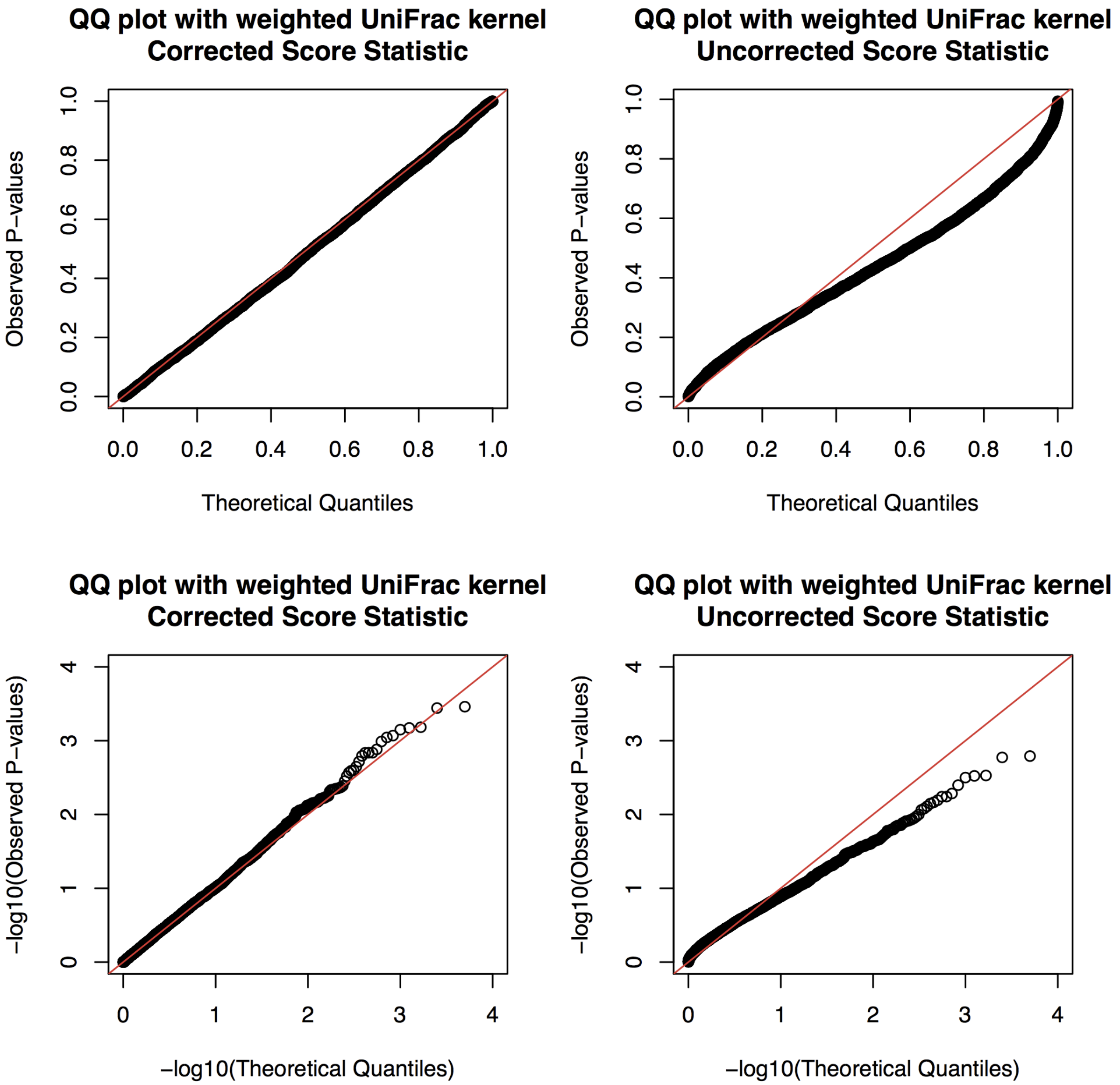

Figure S1: QQ plots demonstrating the performance of the uncorrected and corrected score statistics. The lower two panels display the -$\log_{10}$(P-values) for both statistics plotted against the theoretical distribution, showing that MiRKAT-S with the correction behaves as expected, whereas without the correction, the p-values deviate significantly from the theoretical distribution. The upper two panels provide scientific insight regarding p-values generated using the uncorrected statistic. In particular, true p-values lower than 0.2 will tend to be too large when using the uncorrected statistic, whereas true p-values higher than 0.2 will tend to be too small when using the uncorrected statistic. This is consistent with what was observed in the application of MiRKAT-S to the aGVHD data.

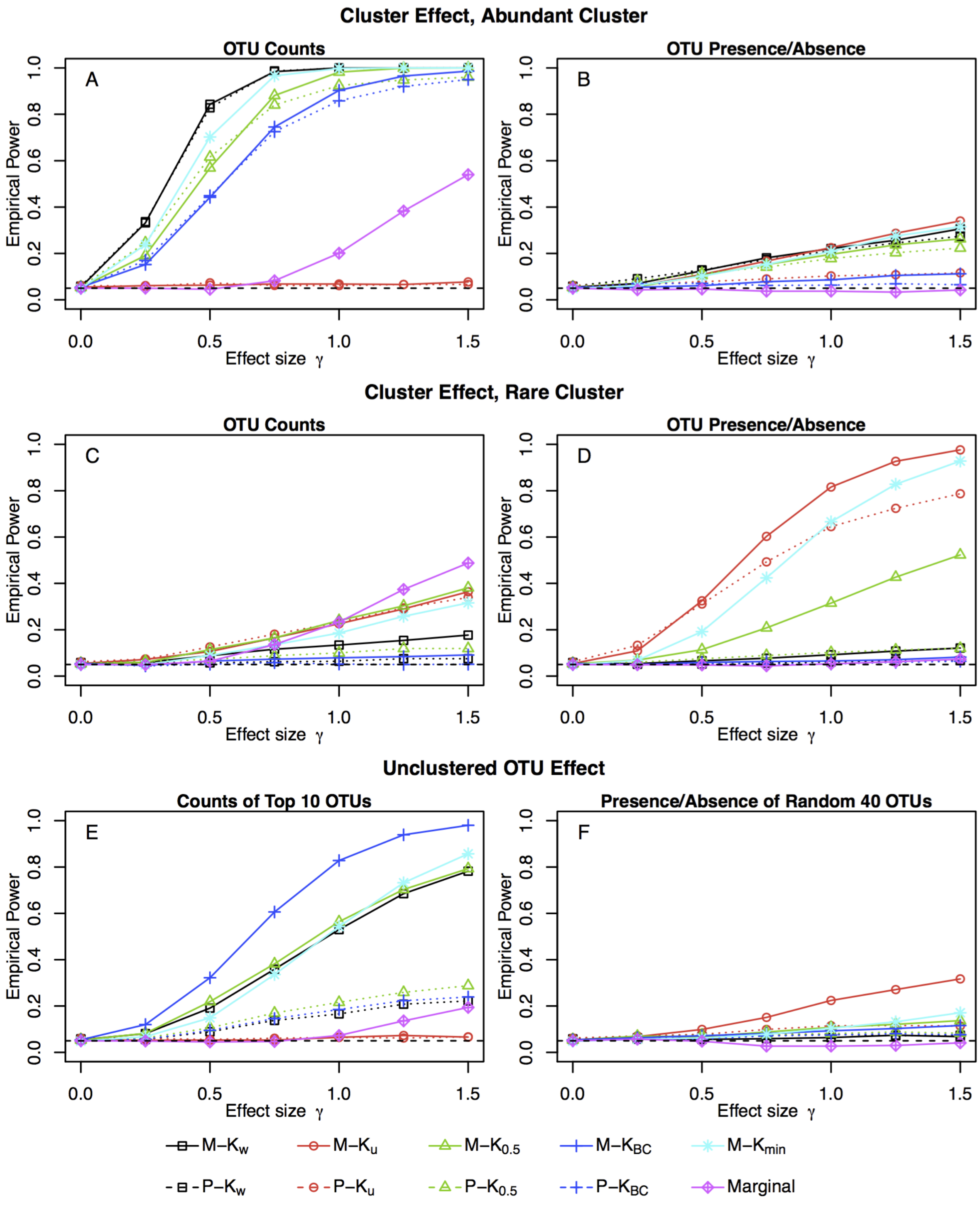
Cluster Effect, Abundant Cluster
OTU Counts
A
OTU Presence/Absence
B
Cluster Effect, Rare Cluster
OTU Counts
C
OTU Presence/Absence
D
Unclustered OTU Effect
Counts of Top 10 OTUs
E
Presence/Absence of Random 40 OTUs
F
Empirical Power
Effect size γ
0.0
0.2
0.4
0.6
0.8
1.0
0.5
1.5
M–Kw
M–Ku
M–K0.5
M–KBC
M–Kmin
P–Kw
P–Ku
P–K0.5
P–KBC
Marginal

Figure S2: Empirical power including MiRKAT-S as in main text, as well as the minimum p-value test, marginal (OTU-level) association analysis, and PCoA. Power was evaluated in all simulation settings, using a sample size of n=100 and 25% censoring. M-K_w, M-K_u, M-K_{BC}, and M-$K_{0.5}$ represent MiRKAT-S results for the weighted UniFrac kernel, unweighted UniFrac kernel, Bray-Curtis kernel, and generalized UniFrac kernel with $\alpha = 0.5$, respectively. P-K_w, P-K_u, P-K_{BC}, and P-$K_{0.5}$ are the corresponding p-values from PCoA using the same set of kernels. M-$K_{\min}$ uses the minimum FDR-adjusted p-value from MiRKAT-S among all four kernels. "Marginal" refers to the permutation p-value summarizing OTU-level tests of all 856 OTUs. γ is the true effect size for the associated cluster or OTUs. (a) Setting 1; survival is associated with OTU counts in a common cluster containing 19.7% of all reads. (b) Setting 3; survival is associated with the presence or absence of each taxon in a common cluster. (c) Setting 1; Survival is associated with OTU counts in a rare cluster containing 0.9% of all reads. (d) Setting 3; survival is associated with the presence or absence of each taxon in a rare cluster. (e) Setting 2; survival is associated with the ten most common OTUs, regardless of cluster membership. (f) Setting 4; survival is associated with forty OTUs selected at random, regardless of cluster membership.
